# Causes of death in Germany: A time series analysis of official statistics from 1990 to 2020

**DOI:** 10.1093/eurpub/ckac130.198

**Published:** 2022-10-25

**Authors:** D Montano

**Affiliations:** Department of Population-Based Medicine, University of Tübingen, Tübingen, Germany

## Abstract

**Background:**

The analysis of the temporal patterns of causes of death is one of the most important tasks in population health monitoring and forecasts. In the present study, a detailed time series analysis of official statistics is performed in order to identify major temporal trends in the distribution of health risks in the German population.

**Methods:**

Official statistics on causes of death from 1990 to 2020 are utilised. The causes of death are classified according to the International Classification of Diseases (10th Revision). Temporal trends of death cases per 100,000 population and ten-year forecasts are estimated with integrated autoregressive moving average models (ARIMA).

**Results:**

Cardiovascular diseases, neoplasms and cerebrovascular diseases have accounted for more than 70% of all deaths between 1990-2020. In contrast, urogenital, infectious and muscular-related diseases have been reported for less than 2% of deaths during the same period. Annual deaths per 100,000 population due to cardiovascular and cerebrovascular diseases largely decreased between 1990-2020 (-11.07 95% CI [-15.17; -6.97] and -4.02 95% CI [-6.85; -1.20], respectively). Concerning other causes of deaths, no temporal trends were observed, with the exception of diseases of the nervous and digestive system (0.83 95% CI [0.08; 1.58]). The forecasts for the decade 2020-2030 suggest that cardiovascular diseases and neoplasms are expected to remain the most frequent causes of death in Germany and could account for about 67% of all deaths.

**Conclusions:**

The results indicate that non-communicable diseases, in particular the group of cardiovascular diseases and neoplasms, will remain the major driver of mortality in Germany over the next decade.

**Key messages:**

- Cardiovascular health has greatly improved over the last decades in Germany.
- Non-communicable diseases are likely to remain the main drivers of morbidity and mortality over the next decade.

